# IL-10 Promotes Neurite Outgrowth and Synapse Formation in Cultured Cortical Neurons after the Oxygen-Glucose Deprivation via JAK1/STAT3 Pathway

**DOI:** 10.1038/srep30459

**Published:** 2016-07-26

**Authors:** Hongbin Chen, Wei Lin, Yixian Zhang, Longzai Lin, Jianhao Chen, Yongping Zeng, Mouwei Zheng, Zezhong Zhuang, Houwei Du, Ronghua Chen, Nan Liu

**Affiliations:** 1 Department of Neurology, The Affiliated Union Hospital, Fujian Medical University, Fuzhou, Fujian, People’s Republic of China; 2 Institute of Cerebral Vascular Disease of Fujian Province, Fuzhou, Fujian, People’s Republic of China; 3 Department of Rehabilitation, The Affiliated Union Hospital, Fujian Medical University, Fuzhou, Fujian, People’s Republic of China

## Abstract

As a classic immunoregulatory and anti-inflammatory cytokine, interleukin-10 (IL-10) provides neuroprotection in cerebral ischemia *in vivo* or oxygen-glucose deprivation (OGD)-induced injury *in vitro*. However, it remains blurred whether IL-10 promotes neurite outgrowth and synapse formation in cultured primary cortical neurons after OGD injury. In order to evaluate its effect on neuronal apoptosis, neurite outgrowth and synapse formation, we administered IL-10 or IL-10 neutralizing antibody (IL-10NA) to cultured rat primary cortical neurons after OGD injury. We found that IL-10 treatment activated the Janus kinase 1 (JAK1)/signal transducers and activators of transcription 3 (STAT3) signaling pathway. Moreover, IL-10 attenuated OGD-induced neuronal apoptosis by down-regulating the Bax expression and up-regulating the Bcl-2 expression, facilitated neurite outgrowth by increasing the expression of Netrin-1, and promoted synapse formation in cultured primary cortical neurons after OGD injury. These effects were partly abolished by JAK1 inhibitor GLPG0634. Contrarily, IL-10NA produced opposite effects on the cultured cortical neurons after OGD injury. Taken together, our findings suggest that IL-10 not only attenuates neuronal apoptosis, but also promotes neurite outgrowth and synapse formation via the JAK1/STAT3 signaling pathway in cultured primary cortical neurons after OGD injury.

As a main cause of death and disability worldwide, stroke typically results in persistent severe neurological impairment^1^. After the cerebral ischemia, the immune system plays a critical role in the activation of immune cells and release of inflammatory cytokines in the subacute phase^1^,^2^,^3^,^4^,^5^. These pathophysiological responses can induce axonal injury, which eventually leads to the reduction or loss of neuronal and synaptic connectivity, contributing to the impairment of neurological function^6^,^7^. Therefore, neurite outgrowth and synaptogenesis are essential for the neuronal functional recovery after the cerebral ischemia^8^,^9^.

As a pivotal anti-inflammatory cytokine, interleukin-10 (IL-10) suppresses immune responses and attenuates local inflammatory reactions and neuronal damages after the cerebral infarction^2^,^10^,^11^. Indeed, in IL-10-deficient-mice, the infarct volume and level of pro-inflammatory cytokines increased and the neurological impairment worsened after the permanent ischemia^10^,^12^. Consistent with these reports, a lower level of IL-10 in patients with cerebral stroke is associated with a worse outcome^13^. In addition, exogenous IL-10, in a concentration-dependent manner, reduces neuronal apoptosis in cultured primary cortical neurons exposed to oxygen-glucose deprivation (OGD)^12^,^14^. A growing body of evidence has documented the favorable neuroprotective effect of IL-10 on cerebral ischemic injury *in vivo* or OGD-induced injury *in vitro*. In a recent study, the administration of IL-10 promotes via the IL-10 receptor the synapse formation in cultured hippocampal neurons^15^. However, little direct evidence is available with regards to the effects of IL-10 on the neurite outgrowth and synapse formation of neurons that are afflicted with OGD-induced injury.

In the IL-10-mediated anti-inflammatory response, the Janus kinase 1 (JAK1)/signal transducers and activators of transcription 3 (STAT3) signaling pathway is an important signal transduction cascade^16^,^17^,^18^. JAK1 is phosphorylated by IL-10 and becomes activated^19^ and in turn phosphorylates STAT3 and makes it active^18^,^20^,^21^. Many studies have documented that activated JAK1 and STAT3 are up-regulated in neurons, astrocytes and microglia after focal cerebral infarction^21^,^22^ and may provide neuroprotection in the acute phase of ischemia^23^,^24^,^25^. Indeed, after the injury of nerve, overexpressed and activated STAT3 has been shown to contribute to neuronal survival and axon regeneration^26^,^27^,^28^, and vascular STAT3 has been found to improve the long-term recovery of neurological function by facilitating angiogenesis and axon outgrowth after the stroke^23^. In addition, activated STAT3 has been found to increase synaptophysin expression, which enhances synapse formation in the hippocampus^29^. Given the facts that IL-10 produces the anti-inflammatory effect through the activation of JAK1/STAT3 pathway and that activated STAT3 contributes to axon outgrowth after nerve impairment and promotes synapse formation in the hippocampus, it would be intriguing and rewarding to investigate whether IL-10 promotes neurite outgrowth and synapse formation in OGD-injured neurons and whether the JAK1/STAT3 pathway is involved in this hypothesized effect.

In the present work, we found that IL-10 not only inhibited apoptosis, but also facilitated neurite outgrowth and promoted synaptogenesis in the cultured primary cortical neurons after OGD injury. In addition, we also demonstrated that the JAK1/STAT3 signaling pathway contributed to the neuroprotective effect of IL-10 on anti-apoptosis, neurite outgrowth and synapse formation in cultured primary cortical neurons after OGD injury.

## Results

### Morphology and Purity of Rat Primary Cortical Neurons

**Figure 1:**
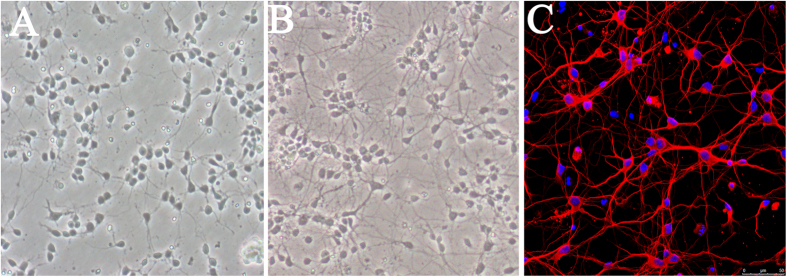
Morphology of cultured primary cortical neurons. (**A**) At day 2, neurons were small with relatively round cell bodies and short neurites (×200). (**B**) At day 7, the neurites of neurons extended and formed a network (×200). (**C**) Immunofluorescence staining of cultured neurons. Cell bodies and neurites were stained with class III-β-Tubulin (red), while nuclei were labeled with Hoechst33342 (blue). Scale bar = 50 μm.

The typical morphology of cultured neurons growing in coverslips was shown in Fig. 1. Two days after culture, neurons were small with relatively round cell bodies and short neurites (Fig. 1A), which extended and formed a network at day 7 (Fig. 1B). The purity of neurons was determined by immunofluorescent staining. Cell bodies and neurites of neurons were stained red with class III-β-Tubulin while the nuclei of all cells were labeled blue with Hoechst33342 (Fig. 1C). The ratio of class III-β-Tubulin-positive cells to all cells was calculated to assess the purity of neurons. The results showed that about 90% of cells were neurons.

### OGD up-regulates the level of IL-10 Receptor in Cultured Primary Cortical Neurons

**Figure 2:**
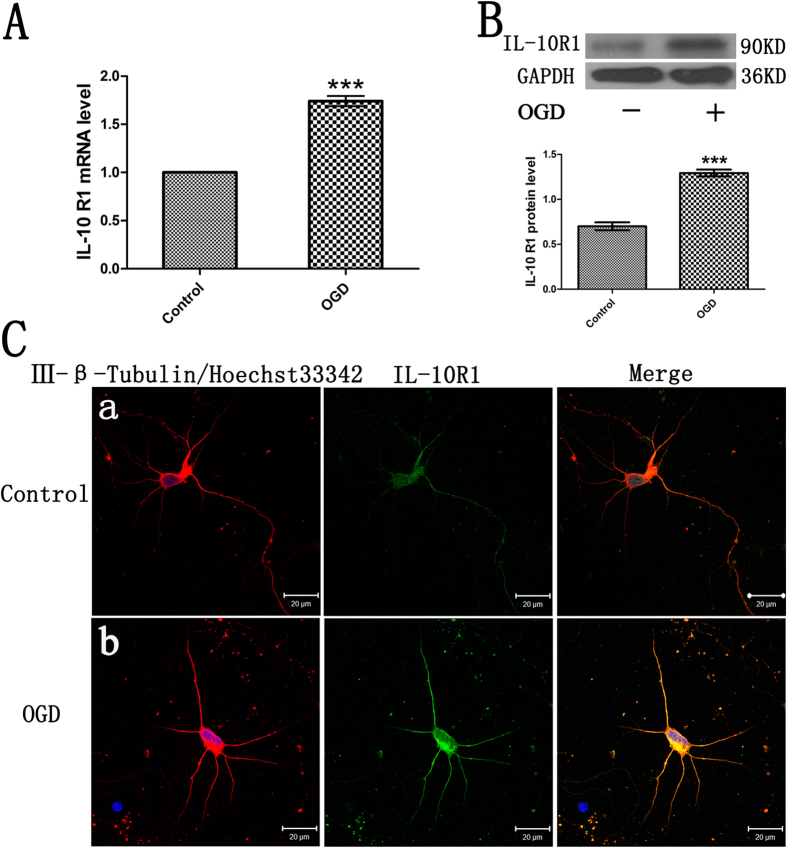
The increased level of IL-10 receptor1 in cultured primary neurons after OGD injury. (**A**) qPCR analysis of IL-10R1 (n = 3). Control group was set as calibrator sample representing the 1 × expression. ***p < 0.001, as compared with the control group; by unpaired two-tailed Student’s t-tests. Data are presented as mean ± SEM. (**B**) Western blot analysis of IL-10R1 (n = 5). ***p < 0.001, as compared with the control group; by unpaired two-tailed Student’s t-tests. Data are presented as mean ± SEM. (**C**) Localization of immunoreactive IL-10R1 expression on neurons before and after OGD. The left column displays neuronal marker class III-β-Tubulin (red) and nucleus (blue). The middle column shows expression of IL-10R1 (green), which was expressed in the cyton and neurites. The right column shows the co-localization of IL-10R1 and class III-β-Tubulin (yellow). Scale bar represents 20 μm.

The IL-10 receptor consists of two subunits IL-10R1 and IL-10R2. After interacting with IL-10R1, IL-10 produces anti-inflammatory actions^18^. To determine whether IL-10 promotes neuronal recovery or repair in cultured cortical neurons after OGD injury, we examined the level of IL-10R1 subunit by qPCR and western blot. qPCR results (Fig. 2A) showed that compared with the control group, the mRNA level of IL-10R1 in the OGD group was markedly increased by 1.74 ± 0.53 (*p* < 0.001), which was further confirmed by western blot. As shown in Fig. 2B, the protein level of IL-10R1 was significantly increased when compared with the control group (1.29 ± 0.38 vs. 0.70 ± 0.44, *p* < 0.001). Immunofluorescence staining was used to detect the presence of IL-10R1 expression in neurons after OGD (Fig. 2C). As shown, immunoreactive IL-10R1 expressed in cyton and neurites were observed by laser confocal microscopy. These results indicate that IL-10R1 level in cultured neurons is up-regulated after OGD.

### IL-10 Activates the JAK1/STAT3 Signal Transductional Pathway in Cultured Primary Cortical Neurons after OGD

**Figure 3:**
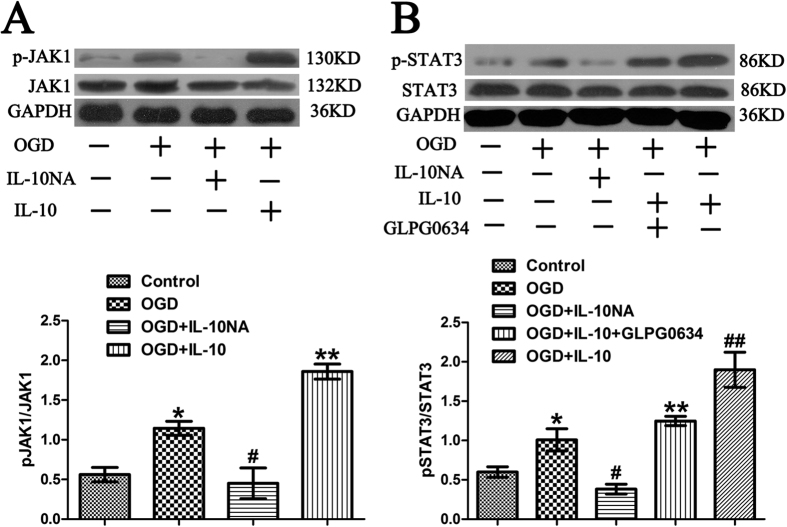
The activation of the JAK1/STAT3 pathway by IL-10 in neurons after OGD. (**A**) Western blot analysis of p-JAK1 (n = 3). *p < 0.05, as compared with control group; ^#^*p* < 0.05, as compared with OGD group; **p < 0.01, as compared with OGD group; by one way analysis of variance (ANOVA) followed by Student-Newman-Keuls multiple comparisons test, F = 26.747, *p* < 0.0001. (**B**) Western blot analysis of p-STAT3 (n = 3). *p < 0.05, as compared with control group; ^#^*p* < 0.05, as compared with OGD group; ^##^*p* < 0.01, as compared with OGD group; **p < 0.01, as compared with OGD + IL-10 group; by one way analysis of variance (ANOVA) followed by Student-Newman-Keuls multiple comparisons test, F = 21.554, *p* < 0.0001. Data are presented as mean ± SEM.

To elucidate whether IL-10 activates JAK1/STAT3 pathway in cultured cortical neurons after OGD, we next analyzed the change of p-JAK1 and p-STAT3 after IL-10 and IL-10NA was respectively administered to the neurons. We found that the expression of p-JAK1 and p-STAT3 in the OGD group was increased when compared with that of the control group (p-JAK1, 1.15 ± 0.09 vs. 0.56 ± 0.09, *p* < 0.05; p-STAT3, 1.01 ± 0.14 vs. 0.60 ± 0.07, *p* < 0.05) (Fig. 3). Meanwhile, compared with the OGD group, the OGD + IL-10 group reported higher expression of p-JAK1 and p-STAT3 (p-JAK1, 1.86 ± 0.09 vs. 1.15 ± 0.09, *p* < 0.01; p-STAT3, 1.90 ± 0.22 vs. 1.01 ± 0.14, *p* < 0.01); however, the expression of p-JAK1 and p-STAT3 was decreased in the OGD + IL-10NA group (p-JAK1, 0.45 ± 0.19 vs. 1.15 ± 0.09, *p* < 0.05; p-STAT3, 0.38 ± 0.06 vs. 1.01 ± 0.14, *p* < 0.05) (Fig. 3). Moreover, after GLPG0634^30^, a specific inhibitor of JAK1, was added to the OGD-injured neurons treated with IL-10, the expression of p-STAT3 was decreased in the OGD + IL-10 + GLPG0634 group when compared with that of the OGD + IL-10 group (1.25 ± 0.06 vs. 1.90 ± 0.22, *p* < 0.01) (Fig. 3B). No significant difference was found between the OGD group and OGD + IL-10 + GLPG0634 group (1.01 ± 0.14 vs. 1.25 ± 0.06, *p* > 0.05) (Fig. 3). These findings suggest that IL-10 activates the JAK1/STAT3 pathway in neurons after OGD, while IL-10NA can block this activation and JAK1 inhibitor GLPG0634 can partly reverse it.

### IL-10 Attenuates OGD-induced Apoptosis in Cultured Primary Cortical Neurons via JAK1/STAT3 Pathway

**Figure 4:**
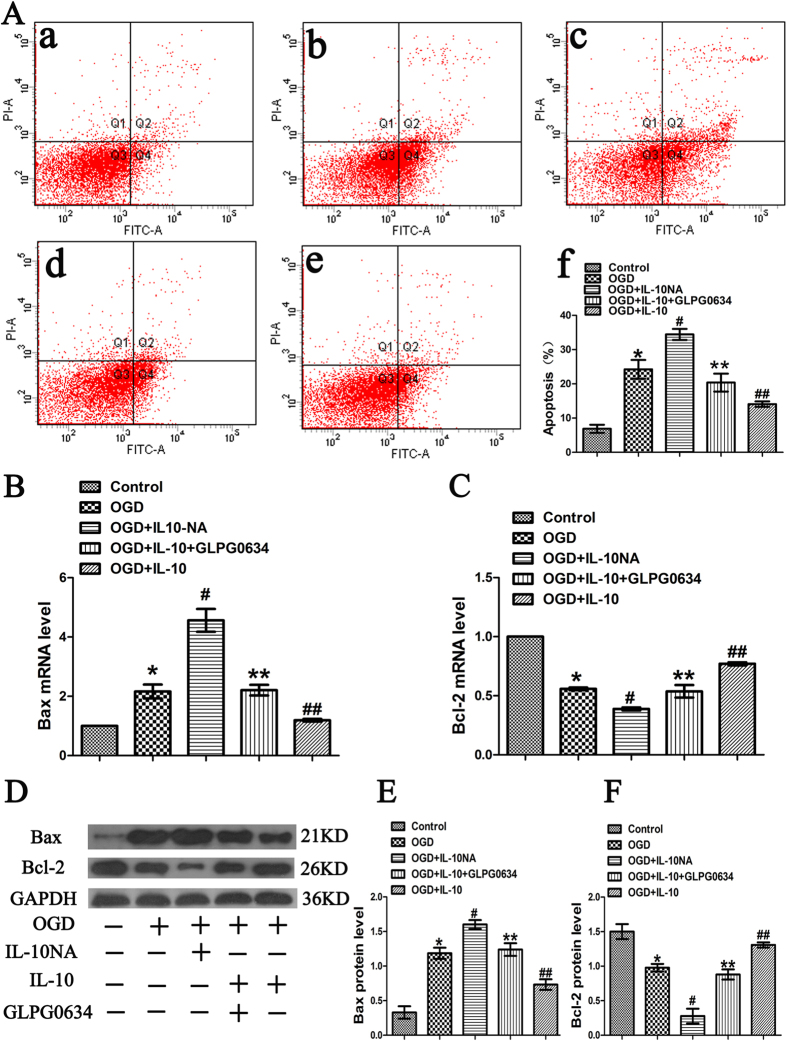
OGD-induced apoptosis attenuated by IL-10 via JAK1/STAT3 pathway. (**A**) Twenty-four hours after OGD, apoptosis of neurons were detected by flow cytometry. The signals from apoptotic neurons were localized in the Q2 and Q4 quadrants of the resulting dot-plot graph. (a) Control group; (b) OGD group; (c) OGD + IL-10NA group; (d) OGD + IL-10 + GLPG0634 group; (e) OGD + IL-10 group (f) Statistical graph of apoptosis in different groups (n = 3). *p < 0.001, as compared with control group; ^#^*p* < 0.01, as compared with OGD group; ^##^*p* < 0.01, as compared with OGD group; **p < 0.05, as compared with OGD + IL-10 group; by one way analysis of variance (ANOVA) followed by Student-Newman-Keuls multiple comparisons test, F = 28.579, *p* < 0.0001. (**B**) qPCR analysis of Bax (n = 4). Control group was set as calibrator sample representing the 1 × expression. *p < 0.01, as compared with control group; ^#^*p* < 0.001, as compared with OGD group; ^##^*p* < 0.05, as compared with OGD group; **p < 0.05, as compared with OGD + IL-10 group; by one way analysis of variance (ANOVA) followed by Student-Newman-Keuls multiple comparisons test, F = 42.490, *p* < 0.0001. (**C**) qPCR analysis of Bcl-2 (n = 3). Control group was set as calibrator sample representing the 1 × expression. *p < 0.001, as compared with control group; ^#^*p* < 0.01, as compared with OGD group; ^##^*p* < 0.001, as compared with OGD group; **p < 0.001, as compared with OGD + IL-10 group; by one way analysis of variance (ANOVA) followed by Student-Newman-Keuls multiple comparisons test, F = 86.127, *p* < 0.0001. (**D**) The representative image of western blot analysis for Bax and Bcl-2 expression. (**E**) Western blot analysis of Bax (n = 4). *p < 0.001, as compared with control group; ^#^*p* < 0.01, as compared with OGD group; ^##^*p* < 0.01, as compared with OGD group; **p < 0.01, as compared with OGD + IL-10 group; by one way analysis of variance (ANOVA) followed by Student-Newman-Keuls multiple comparisons test, F = 36.711, *p* < 0.0001. (**F**) Western blot analysis of Bcl-2 (n = 4). *p < 0.01, as compared with control group; ^#^*p* < 0.001, as compared with OGD group; ^##^*p* < 0.05, as compared with OGD group; **p < 0.01, as compared with OGD + IL-10 group; by one way analysis of variance (ANOVA) followed by Student-Newman-Keuls multiple comparisons test, F = 33.323, *p* < 0.0001. All data are presented as mean ± SEM.

In order to evaluate the effect of IL-10 on apoptosis, we treated neurons with IL-10 or IL-10NA after OGD. To further investigate whether JAK1/STAT3 pathway is involved in anti-apoptotic effect of IL-10, GLPG0634 was used to interrupt the phosphorylation of JAK1 specifically. Results from flow cytometry assay showed that OGD significantly increased the apoptosis when compared with the control group (24.23 ± 2.74% vs. 6.87 ± 1.19%, *p* < 0.001) (Fig. 4A). Moreover, as indicated in Fig. 4A, compared with that of the OGD group, the apoptosis rate was lowered in the OGD + IL-10 group but markedly increased in the OGD + IL-10NA group (14.10 ± 0.80% vs. 24.23 ± 2.74%, 34.43 ± 1.63% vs. 24.23 ± 2.74%, *p* < 0.01, respectively). Compared with the OGD + IL-10 group, OGD + IL-10 + GLPG0634 group reported a much higher apoptosis rate (20.37 ± 2.60% vs. 14.10 ± 0.80%, *p* < 0.05) but no significant difference was found between OGD group and OGD + IL-10 + GLPG0634 group (24.23 ± 2.74% vs. 20.37 ± 2.60%, *>* 0.05).

To further elucidate the molecular mechanism by which IL-10 exerts an anti-apoptotic effect on neurons after OGD, we analyzed the expression of Bax and Bcl-2 by qPCR and Western blot. qPCR results showed that when compared with those of the control group, OGD increased Bax expression (2.16 ± 0.23, *p* < 0.01) (Fig. 4B) but decreased Bcl-2 expression (0.56 ± 0.01, *p* < 0.001) (Fig. 4C). When compared with those of OGD group, IL-10 treatment reduced Bax expression and increased Bcl-2 expression in OGD-injured neurons (Bax, 1.19 ± 0.05 vs. 2.16 ± 0.23, *p* < 0.05; Bcl-2, 0.77 ± 0.01 vs. 0.56 ± 0.01, *p* < 0.001) (Fig. 4B,C); contrarily, IL-10NA treatment significantly increased Bax expression and decreased Bcl-2 expression (Bax, 4.56 ± 0.38 vs. 2.16 ± 0.23, *p* < 0.001; Bcl-2, 0.38 ± 0.01 vs. 0.56 ± 0.01, *p* < 0.01) (Fig. 4B,C). Additionally, the effects of IL-10 on the expression of Bax and Bcl-2 were reversed by GLPG0634 (Bax, 2.21 ± 0.18 vs. 1.19 ± 0.05, *p* < 0.05; Bcl-2, 0.54 ± 0.05 vs. 0.77 ± 0.01, *p* < 0.001) (Fig. 4B,C). Consistent with these results, western blot results indicated that neurons exposed to OGD showed higher expression of Bax, but lower expression of Bcl-2 when compared with the control group (Bax, 1.19 ± 0.08 vs. 0.33 ± 0.09, *p* < 0.001; Bcl-2, 0.98 ± 0.06 vs. 1.50 ± 0.11, *p* < 0.01) (Fig. 4E,F). When compared with OGD group, OGD + IL-10 group displayed a significant decrease in Bax expression and a marked increase in Bcl-2 expression (0.73 ± 0.08 vs. 1.19 ± 0.08, *p* < 0.01; 1.31 ± 0.04 vs. 0.98 ± 0.06, *p* < 0.05, respectively) (Fig. 4E,F); OGD + IL-10NA group reported a higher Bax expression but a lower Bcl-2 expression (1.60 ± 0.06 vs. 1.19 ± 0.08, *p* < 0.01; 0.28 ± 0.11 vs. 0.98 ± 0.06, *p* < 0.001, respectively) (Fig. 4E,F). Moreover, compared with the OGD + IL-10 group, OGD + IL-10 + GLPG0634 group induced a much higher Bax expression but a lower Bcl-2 expression (Bax, 1.24 ± 0.09 vs. 0.73 ± 0.08, *p* < 0.01; Bcl-2, 0.88 ± 0.07 vs. 1.31 ± 0.04, *p* < 0.01) (Fig. 4E,F). These results demonstrate that IL-10 attenuates OGD-induced apoptosis in neurons by down-regulating the expression of pro-apoptotic protein Bax and up-regulating the expression of the anti-apoptotic protein Bcl-2. This anti-apoptotic effect can be partially blocked by JAK1 inhibitor GLPG0634. Furthermore, IL-10NA increases neuron apoptosis after OGD by increasing Bax expression and decreasing Bcl-2 expression.

### IL-10 Facilitates Neurite Outgrowth in Cultured Primary Cortical Neurons after OGD Injury via JAK1/STAT3/Netrin-1 Pathway

**Figure 5:**
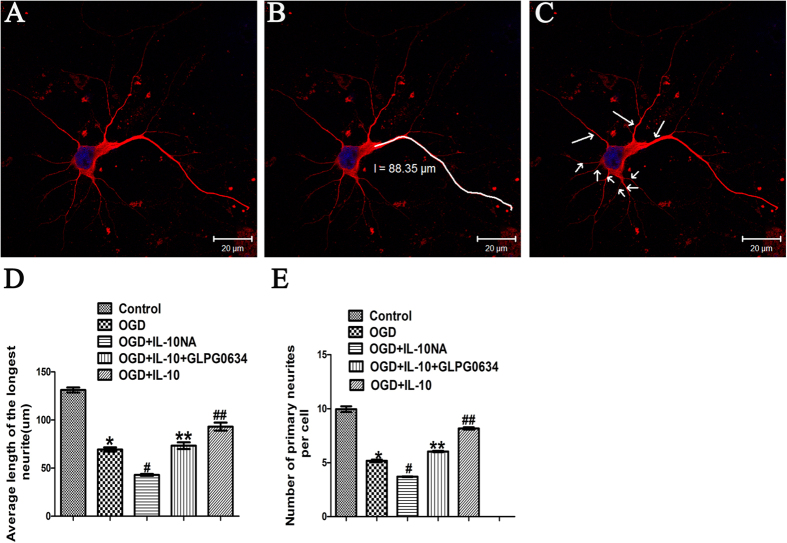
Detection of neurites by immunofluorescence. (**A**) Cyton, neurites (red) and nucleus (blue). (**B**) Manually tracing the length of the longest neurite (white) with the LSM (4.2.0.121) software. (**C**) Number of primary neurites (arrows) was calculated. (**D**) Statistical analysis of the longest neurite length (n = 3). *p < 0.001, as compared with control group; ^#^*p* < 0.001, as compared with OGD group; ^##^*p* < 0.001, as compared with OGD group; **p < 0.001, as compared with OGD + IL-10 group; by one way analysis of variance (ANOVA) followed by Student-Newman-Keuls multiple comparisons test, F = 128.113, *p* < 0.0001. (**E**) Statistical analysis of the number of primary neurites per cell (n = 3). *p < 0.001, as compared with control group; ^#^*p* < 0.001, as compared with OGD group; ^##^*p* < 0.001, as compared with OGD group; **p < 0.001, as compared with OGD + IL-10 group; by one way analysis of variance (ANOVA) followed by Student-Newman-Keuls multiple comparisons test, F = 305.781, *p* < 0.0001. For each group and experiment, 20 neurons from 8 ~ 10 randomly selected fields were observed to assess the average length of the longest neurites and average number of primary neurites. Data are presented as mean ± SEM of three independent experiments (n = 3). Scale bar: 20 μm.

**Figure 6:**
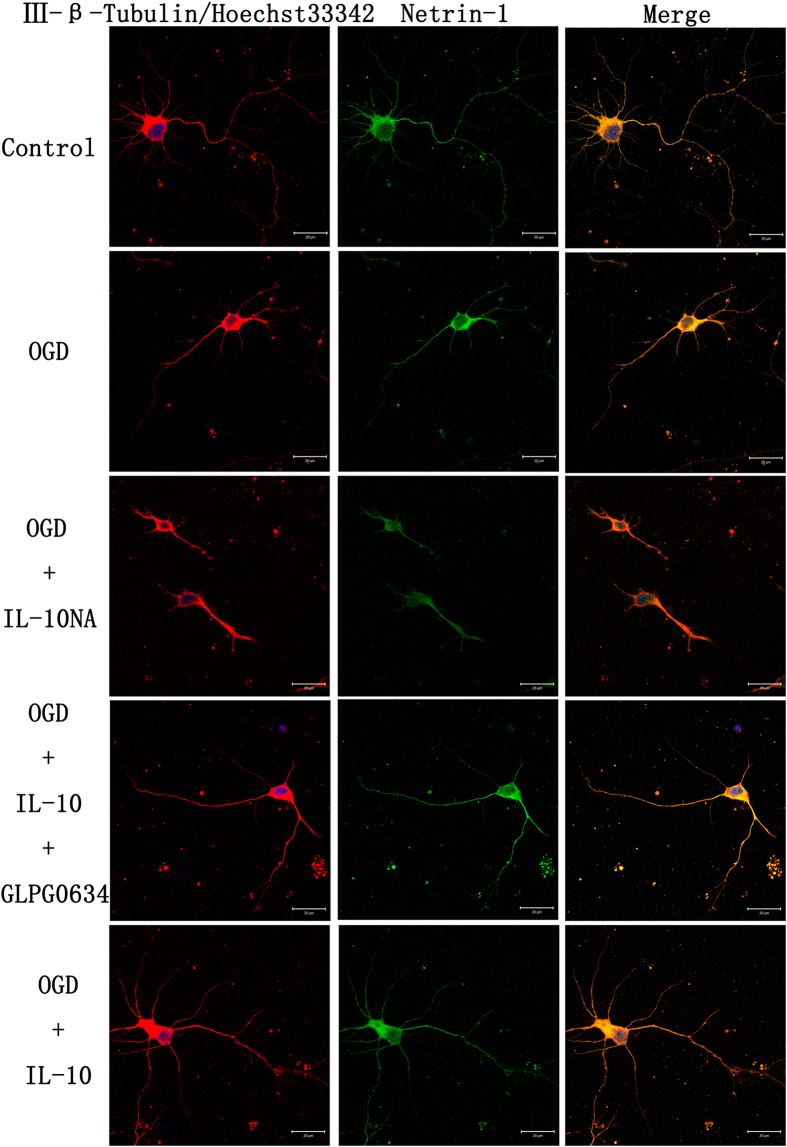
Effect of IL-10 on neurite outgrowth and localization of Netrin-1 expression. Representative images of each group were shown. The left column displays neuronal marker class III-β-Tubulin (red) and nucleus (blue). The length of the longest neurites and the number of primary neurites were counted. The middle column shows expression of Netrin-1 (green), which was expressed in the cyton and neurites. The right column shows the co-localization of Netrin-1 and class III-β-Tubulin (yellow). Scale bar represents 20 μm.

To elucidate the direct effect of IL-10 on neurite outgrowth, we exposed the neurons to IL-10 or IL-10NA after OGD. Then the average length of the longest neurites and average number of primary neurites were calculated. As shown in Figs 5D and 6, when compared with the control group, in the OGD group, most neurites were ruptured and shortened, the average length of the longest neurites was markedly decreased (69.37 ± 2.14 μm vs. 131.20 ± 2.63 μm, *p* < 0.001) and the number of primary neurites was significantly decreased (5.17 ± 0.12 vs. 9.95 ± 0.26, *p* < 0.001) (Figs 5E and 6). When compared with that of the OGD group, the average length of the longest neurites was significantly increased in OGD + IL-10 group (93.12 ± 4.17 μm vs. 69.37 ± 2.14 μm, *p* < 0.001), but markedly decreased in OGD + IL-10NA group (42.94 ± 0.94 μm vs. 69.37 ± 2.14 μm, *p* < 0.001) (Figs 5D and 6). Consistent with these results, the number of primary neurites was also increased in OGD + IL-10 group (8.17 ± 0.10 vs. 5.17 ± 0.12, *p* < 0.001), but decreased in OGD + IL-10NA group (3.70 ± 0.03 vs. 5.17 ± 0.12, *p* < 0.001) (Figs 5E and 6). To further ascertain whether JAK1/STAT3 pathway is involved in the effect of IL-10 on neurite outgrowth, GLPG0634 was used to interrupt the phosphorylation of JAK1 specifically. The results showed that OGD + IL-10 + GLPG0634 group exhibited shorter neurite length and fewer primary neurites when compared with OGD + IL-10 group (73.30 ± 3.50 μm vs. 93.12 ± 4.17 μm, *p* < 0.001; 6.03 ± 0.07 vs. 8.17 ± 0.10, *p* < 0.001, respectively) (Figs 5 and 6). These data suggest that IL-10 facilitates neurite outgrowth in cultured primary cortical neurons after OGD injury and that IL-10-induced neurite outgrowth is partly reversed by JAK1 inhibitor GLPG0634. Similar to the effect of GLPG0634, IL-10NA restrains neurite outgrowth after OGD injury.

**Figure 7:**
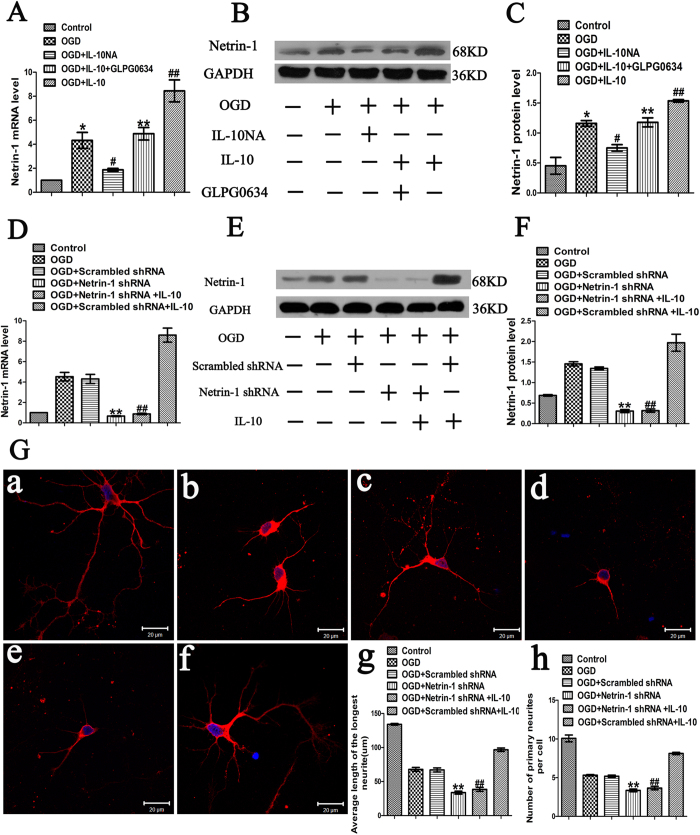
The effect of IL-10 on neurite outgrowth mediated by Netrin-1 after OGD injury. (**A**) qPCR analysis of Netrin-1 (n = 3). Control group was set as calibrator sample representing the 1× expression. *p < 0.01, as compared with control group; ^#^*p* < 0.05, as compared with OGD group; ^**##**^*p* < 0.01, as compared with OGD group; **p < 0.01, as compared with OGD + IL-10 group; by one way analysis of variance (ANOVA) followed by Student-Newman-Keuls multiple comparisons test, F = 27.274, *p* < 0.0001. (**B**) The representative image of western blot analysis for Netrin-1. (**C**) Western blot analysis of Netrin-1 (n = 3). *p < 0.001, as compared with control group; ^#^*p* < 0.01, as compared with OGD group; ^##^*p* < 0.05, as compared with OGD group; **p < 0.05, as compared with OGD + IL-10 group; by one way analysis of variance (ANOVA) followed by Student-Newman-Keuls multiple comparisons test, F = 28.529, *p* < 0.0001. (**D**) qPCR analysis of the knockdown efficiency of Netrin-1 (n = 3). Control group was set as calibrator sample representing the 1 × expression. **p < 0.001, as compared with OGD + Scrambled shRNA group; ^**##**^*p* < 0.001, as compared with OGD + Scrambled shRNA + IL-10 group; by one way analysis of variance (ANOVA) followed by Student-Newman-Keuls multiple comparisons test, F = 109.194, *p* < 0.0001. (**E**) The representative image of western blot analysis of the knockdown efficiency of Netrin-1. (**F**) Western blot analysis of the knockdown efficiency of Netrin-1 (n = 5). **p < 0.001, as compared with OGD + Scrambled shRNA group; ^**##**^*p* < 0.001, as compared with OGD + Scrambled shRNA + IL-10 group; by one way analysis of variance (ANOVA) followed by Student-Newman-Keuls multiple comparisons test, F = 173.098, *p* < 0.0001. (**G**) Detection of neurites after Netrin-1 knockdown. (a) Control group; (b) OGD group; (c) OGD + scrambled shRNA group; (d) OGD + Netrin-1 shRNA group; (e) OGD + Netrin-1 shRNA + IL-10 group; (f) OGD + scrambled shRNA + IL-10 group; (g) Statistical analysis of the longest neurite length after Netrin-1 knockdown (n = 3). **p < 0.001, as compared with OGD + Scrambled shRNA group; ^**##**^*p* < 0.001, as compared with OGD + Scrambled shRNA + IL-10 group; by one way analysis of variance (ANOVA) followed by Student-Newman-Keuls multiple comparisons test, F = 217.416, *p* < 0.0001. (h) Statistical analysis of average number of primary neurites after Netrin-1 knockdown (n = 3). **p < 0.001, as compared with OGD + Scrambled shRNA group; ^**##**^*p* < 0.001, as compared with OGD + Scrambled shRNA + IL-10 group; by one way analysis of variance (ANOVA) followed by Student-Newman-Keuls multiple comparisons test, F = 130.618, *p* < 0.0001. Scale bar: 20 μm. Data are presented as mean ± SEM.

To explore the molecular mechanism by which IL-10 promotes neurite outgrowth on neurons after OGD injury, we analyzed the expression of Netrin-1 by qPCR and Western blot. qPCR results demonstrated that OGD markedly increased the expression of Netrin-1 (4.32 ± 0.67, *p* < 0.01) (Fig. 7A); moreover, when compared with that of the OGD group, the expression of Netrin-1 of the OGD + IL-10 group was obviously increased (8.45 ± 0.91 vs. 4.32 ± 0.67, *p* < 0.01) (Fig. 7A); and that of the OGD + IL-10NA group was significantly lowered (1.87 ± 0.11 vs. 4.32 ± 0.67, *p* < 0.05) (Fig. 7A). To further ascertain whether IL-10 regulates the expression of Netrin-1 by the JAK1/STAT3 pathway, GLPG0634 was added into neurons treated with IL-10 to interrupt the phosphorylation of JAK1 specifically. The results confirmed that OGD + IL-10 + GLPG0634 group showed a lower expression of Netrin-1 when compared with the OGD + IL-10 group (4.88 ± 0.52 vs. 8.45 ± 0.91, *p* < 0.01) (Fig. 7A). Consistent with these results, western blot indicated that OGD increased the expression of Netrin-1 (1.16 ± 0.05 vs. 0.45 ± 0.14, *p* < 0.001) (Fig. 7C); furthermore, compared with the OGD group, the OGD + IL-10 group presented higher protein expression of Netrin-1 (1.54 ± 0.02 vs. 1.16 ± 0.05, *p* < 0.05) (Fig. 7C); however, when exposed to IL-10NA, OGD-injured neurons showed lower expression of Netrin-1 in comparison with that of the OGD group (0.75 ± 0.05 vs. 1.16 ± 0.05, *p* < 0.01); and the expression of Netrin-1 was reduced in OGD + IL-10 + GLPG0634 group when compared with that of the OGD + IL-10 group (1.18 ± 0.07 vs. 1.54 ± 0.02, *p* < 0.05) (Fig. 7C). These results demonstrate that IL-10 up-regulates Netrin-1 expression and that these effects can be partly neutralized by JAK1 inhibitor GLPG0634. In contrast to IL-10, IL-10NA suppresses the expression of Netrin-1.

To ascertain the role of Netrin-1 expression in neurite outgrowth, we evaluated the outgrowth in IL-10 treatment after the Netrin-1 knockdown using shRNA. The experimental groups were designed as follows: control group, OGD group, and OGD + scrambled shRNA group, OGD + Netrin-1 shRNA group, OGD + Netrin-1 shRNA + IL-10 group, OGD + scrambled shRNA + IL-10 group. The knockdown efficiency of Netrin-1 was determined by qPCR and western blot. As shown in Fig. 7D, the mRNA level of Netrin-1 in the OGD + Netrin-1 shRNA group was significantly lower than that of the OGD + scrambled shRNA group (0.67 ± 0.05 vs. 4.31 ± 0.44, *p* < 0.001); moreover, the mRNA level of Netrin-1 in the OGD + Netrin-1 shRNA + IL-10 group was obviously decreased when compared with that of the OGD + scrambled shRNA + IL-10 group (0.88 ± 0.08 vs. 8.60 ± 0.40, *p* < 0.001). Consistent with these results, western blot indicated that the protein level of Netrin-1 in the OGD + Netrin-1 shRNA group was significantly lower than that of the OGD + scrambled shRNA group (0.31 ± 0.04 vs. 1.34 ± 0.04, *p* < 0.001) (Fig. 7F); likewise, compared with the OGD + scrambled shRNA + IL-10 group, the OGD + Netrin-1 shRNA + IL-10 group presented obviously lower protein expression of Netrin-1 (0.32 ± 0.04 vs. 1.97 ± 0.09, *p* < 0.001) (Fig. 7F). Then immunofluorescence staining was used to calculate the average length of the longest neurites and average number of primary neurites. As shown in Fig. 7G, when compared with the OGD + scrambled shRNA group, both the average length of the longest neurites and the number of primary neurites in the OGD + Netrin-1 shRNA group were markedly decreased (33.78 ± 2.22 μm vs. 67.07 ± 2.96 μm, *p* < 0.001; 3.35 ± 0.17 vs. 5.18 ± 0.14, *p* < 0.001, respectively). Compared with the OGD + scrambled shRNA + IL-10 group, the OGD + Netrin-1 shRNA + IL-10 group exhibited shorter neurite length and fewer primary neurites (38.60 ± 2.98 μm vs. 96.74 ± 2.60 μm, *p* < 0.001; 3.65 ± 0.23 vs. 8.12 ± 0.15, *p* < 0.001, respectively) (Fig. 7G). These results indicate that the knockdown of Netrin-1 results in the decrease of neurite outgrowth in OGD-injured neurons and restrains the effect of IL-10 on neurite outgrowth after OGD injury.

### IL-10 promotes synapse formation by JAK1/STAT3 pathway

**Figure 8:**
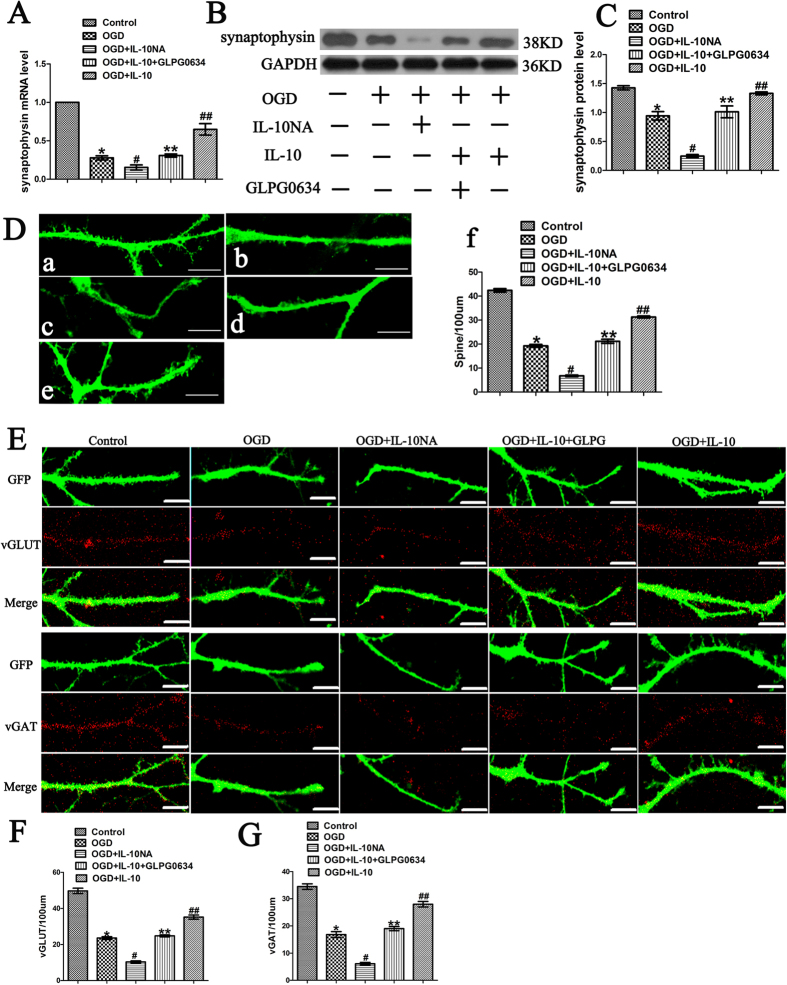
Synapse formation promoted by IL-10 via JAK1/STAT3 pathway. (**A**) qPCR analysis of synaptophysin (n = 3). Control group was set as calibrator sample representing the 1 × expression. *p < 0.001, as compared with control group; ^#^*p* < 0.05, as compared with OGD group; ^##^*p* < 0.001, as compared with OGD group; **p < 0.001, as compared with OGD + IL-10 group; by one way analysis of variance (ANOVA) followed by Student-Newman-Keuls multiple comparisons test, F = 76.701, *p* < 0.0001. (**B**) The representative image of western blot analysis for synaptophysin expression. (**C**) Western blot analysis of synaptophysin (n = 3). *p < 0.01, as compared with control group; ^#^*p* < 0.001, as compared with OGD group; ^##^*p* < 0.01, as compared with OGD group; **p < 0.01, as compared with OGD + IL-10 group; by one way analysis of variance (ANOVA) followed by Student-Newman-Keuls multiple comparisons test, F = 57.656, *p* < 0.0001. (**D**) Statistical analysis of the density of dendritic spine (n = 3). *p < 0.001, as compared with control group; ^#^*p* < 0.001, as compared with OGD group; ^##^*p* < 0.001, as compared with OGD group; **p < 0.001, as compared with OGD + IL-10 group; by one way analysis of variance (ANOVA) followed by Student-Newman-Keuls multiple comparisons test, F = 468.104, *p* < 0.0001. (**E**) The representative image of excitatory synapses and inhibitory synapses for each group. (**F**) Statistical analysis of the density of excitatory synapses (n = 3). *p < 0.001, as compared with control group; ^#^*p* < 0.001, as compared with OGD group; ^##^*p* < 0.001, as compared with OGD group; **p < 0.001, as compared with OGD + IL-10 group; by one way analysis of variance (ANOVA) followed by Student-Newman-Keuls multiple comparisons test, F = 227.982, *p* < 0.0001. (**G**) Statistical analysis of the density of inhibitory synapses (n = 3). *p < 0.001, as compared with control group; ^#^*p* < 0.001, as compared with OGD group; ^##^*p* < 0.001, as compared with OGD group; **p < 0.001, as compared with OGD + IL-10 group; by one way analysis of variance (ANOVA) followed by Student-Newman-Keuls multiple comparisons test, F = 144.251, *p* < 0.0001. For each group and experiment, the density of dendritic spines, excitatory synapses and inhibitory synapses were measured from 20 dendrites of eight to ten neurons. Data are presented as mean ± SEM of three independent experiments (n = 3). Scale bar: 10 μm.

To determine whether IL-10 promotes synapse formation in cultured cortical neurons after OGD injury, we analyzed the expression of synaptophysin and the density of dendritic spines, excitatory and inhibitory synapses. The expression of synaptophysin was detected by qPCR and western blot. qPCR results demonstrated that OGD significantly decreased synaptophysin expression (0.28 ± 0.03, *p* < 0.001, respectively) (Fig. 8A); moreover, when compared with that of the OGD group, the expression of synaptophysin of the OGD + IL-10 group was obviously increased (0.65 ± 0.07 vs. 0.28 ± 0.03, *p* < 0.001) (Fig. 8A); and that of the OGD + IL-10NA group was significantly lowered (0.15 ± 0.03 vs. 0.28 ± 0.03, *p* < 0.05). To further ascertain whether IL-10 regulates the expression of synaptophysin by the JAK1/STAT3 pathway, GLPG0634 was added into neurons treated with IL-10 to interrupt the phosphorylation of JAK1 specifically. The results confirmed that OGD + IL-10 + GLPG0634 group showed a lower expression of synaptophysin when compared with the OGD + IL-10 group (0.31 ± 0.02 vs. 0.65 ± 0.07, *p* < 0.001) (Fig. 8A). Consistent with these results, western blot indicated that OGD decreased synaptophysin expression (0.94 ± 0.07 vs. 1.43 ± 0.04, *p* < 0.01) (Fig. 8C); furthermore, compared with the OGD group, the OGD + IL-10 group presented higher protein expression of synaptophysin (1.33 ± 0.03 vs. 0.94 ± 0.07, *p* < 0.01) (Fig. 8C); however, when exposed to IL-10NA, OGD-injured neurons showed lower expression of Netrin-1 and synaptophysin in comparison with that of the OGD group (0.25 ± 0.03 vs. 0.94 ± 0.07, *p* < 0.001); and the expression of synaptophysin was reduced in OGD + IL-10 + GLPG0634 group when compared with the OGD + IL-10 group (1.01 ± 0.10 vs. 1.33 ± 0.03, *p* < 0.01) (Fig. 8C). These results suggest that IL-10 up-regulates synaptophysin expression and that these effects can be partly neutralized by JAK1 inhibitor GLPG0634. In contrast to IL-10, IL-10NA suppresses the expression of synaptophysin.

To examine the density of dendritic spine on cortical neurons after OGD injury, the cultured cortical neurons were stained with anti-GFP antibody. For the analysis of the density of excitatory and inhibitory synapses, the cultured cortical neurons were stained with anti-vGLUT or anti-vGAT antibody. The results showed that OGD significantly decreased the density of the dendritic spines when compared with that of the control group (19.25 ± 0.60 vs. 42.38 ± 0.73, *p* < 0.001) (Fig. 8D). Indeed, OGD markedly decreased the density of excitatory and inhibitory synapses at the same time (23.58 ± 0.76 vs. 49.70 ± 1.49, *p* < 0.001; 16.82 ± 1.09 vs. 34.47 ± 1.02, *p* < 0.001, respectively) (Fig. 8E–G); moreover, when compared with the OGD group, the density of dendritic spines, excitatory and inhibitory synapses of the OGD + IL-10 group was obviously increased (31.28 ± 0.44 vs. 19.25 ± 0.60, *p* < 0.001; 35.17 ± 1.14 vs. 23.58 ± 0.76, *p* < 0.001; 27.98 ± 1.02 vs. 16.82 ± 1.09, *p* < 0.001, respectively) (Fig. 8D–G); and that of the OGD + IL-10NA group was significantly lowered (6.75 ± 0.36 vs. 19.25 ± 0.60, *p* < 0.001; 10.33 ± 0.57 vs. 23.58 ± 0.76, *p* < 0.001; 6.08 ± 0.49 vs. 16.82 ± 1.09, *p* < 0.001, respectively) (Fig. 8D–G). To further ascertain whether IL-10 regulates the density of dendritic spines, excitatory and inhibitory synapses by the JAK1/STAT3 pathway, we added GLPG0634 to IL-10-treated neurons to interrupt the phosphorylation of JAK1 specifically. The results confirmed that OGD + IL-10 + GLPG0634 group showed a lower density of dendritic spines, excitatory and inhibitory synapses when compared with the OGD + IL-10 group (21.17 ± 0.84 vs. 31.28 ± 0.44, *p* < 0.001; 24.78 ± 0.55 vs. 35.17 ± 1.14, *p* < 0.001; 19.00 ± 0.77 vs. 27.98 ± 1.02, *p* < 0.001, respectively) (Fig. 8D–G). These results indicate that IL-10 increases the density of dendritic spines, excitatory and inhibitory synapses and that these effects can be partly reversed by JAK1 inhibitor GLPG0634. Similar to the effect of GLPG0634, IL-10NA decreases the density of dendritic spines, excitatory and inhibitory synapses.

## Discussion

In this study, we demonstrated that the expression of IL-10 receptor1 (IL-10R1) was increased after OGD injury and that IL-10 activated the JAK1/STAT3 pathway in OGD-injured neurons. The study also found that IL-10 attenuated OGD-induced neuronal apoptosis of cultured primary cortical neurons by down-regulating the Bax expression and up-regulating the Bcl-2 expression, facilitated neurite outgrowth by increasing the expression of Netrin-1, and promoted synapse formation in cultured primary cortical neurons after OGD injury. These effects were partly abolished by JAK1 inhibitor GLPG0634. Contrarily, IL-10NA, the IL-10 neutralizing antibody, produced opposite effects on the cultured cortical neurons after OGD injury.

It has been reported that IL-10 receptor was expressed in cultured cortical neurons^31^. Besides, the expression of IL-10 receptor was strongly up-regulated in the ischemic tissue of wild-type mice after permanent middle cerebral artery occlusion^10^. Adding to existing findings, we provided direct evidence to support that the level of IL-10R1 in cultured cortical neurons was up-regulated after OGD injury. Moreover, we found that immunoreactive IL-10R1 can be detected in cytons and neurites. These findings indicate that OGD-injured neurons are intensively receptive to IL-10, which can directly induce neuroprotective effects on these neurons.

As a classic immunoregulatory and anti-inflammatory cytokine, IL-10 induces neuroprotection in cerebral stroke *in vivo* or OGD-induced injury *in vitro*^12^,^14^,^32^,^33^. After ischemic stroke, it can reduce the infarct volume by 40% in the IL-10 transgenic mice with overexpressed IL-10 in astrocytes, microglia, and endothelial brain cells when compared with that of the wild type mice^33^. In addition, exogenous IL-10, in a concentration-dependent manner, reduces neuronal apoptosis in cultured primary cortical neurons exposed to oxygen-glucose deprivation^12^,^14^. One of the multiple functions of IL-10 is the participation of its signaling pathway in its neuroprotective effects through the receptor-mediated mechanism. When IL-10 combines IL-10R1, it activates the JAK1/STAT3 pathway, which is an important signal transduction cascade that mediates its anti-inflammatory effect^17^,^18^,^34^. A large body of evidence has shown that when phosphorylated by the receptor-associated JAKs^35^, activated STAT3 shows neuroprotective effects by inhibiting apoptosis^36^,^37^,^38^ and providing trophic support for neurons to survive in the acute cerebral ischemia^38^. Our results are in line with and further extend the existing findings, demonstrating that IL-10 activates the JAK1/STAT3 pathway in OGD-injured neurons. Moreover, IL-10 reduces OGD-induced apoptosis in neurons by down-regulating the expression of pro-apoptotic protein Bax and up-regulating that of anti-apoptotic protein Bcl-2. Of note, JAK1 inhibitor GLPG0634 increases neuron apoptosis by preventing IL-10-reduced Bax expression and IL-10-increased Bcl-2 expression. This indicates the involvement of JAK1/STAT3 pathway in the anti-apoptotic effect of IL-10. In contrary to the effects of IL-10, IL-10NA increases OGD-induced apoptosis by inducing Bax expression but suppressing Bcl-2 expression. This result further confirms that IL-10 exerts an anti-apoptotic effect on cultured cortical neurons after OGD injury. As a pro-apoptotic protein, Bax plays a pivotal role in the activation of mitochondrial apoptosis in neurons after transient cerebral ischemia^39^,^40^ while Bcl-2 is crucial for reducing neuron apoptosis in cerebral ischemia by maintaining the stabilization mitochondria membrane potential^41^,^42^,^43^. Taken together, we disclose that IL-10 remarkably reduces OGD-induced apoptosis in neurons by activating the JAK1/STAT3 pathway to down-regulate Bax expression and up-regulate Bcl-2 expression.

After suffering from cerebral ischemic injury, neurites may shrink and decrease^7^. Furthermore, after the loss of their secondary or higher-order dendrites, injured neurons also lose their ability to communicate with other neurons, resulting in neuronal dysfunction or even cell death^44^. However, the dendritic alteration is reversible in injured neurons under certain conditions. According to a previous study, in the early phase, when suffering from an injury which results in dendritic changes, neurons may retain the potential to survive and injury-induced dendritic degeneration can be blocked from proceeding^44^. These suggest that neurites are crucial for neuronal survival after the incidence of CNS injury and that efforts should be spent on the neurite outgrowth, which is essential for regeneration of neuronal networks and neuronal functional recovery after CNS injury^9^,^45^. As a widely used cerebral ischemic injury model *in vitro*^46^,^47^,^48^,^49^, OGD significantly decreases the neurite length and density of cultured neurons^50^. In agreement with the finding, we found that cultured primary cortical neurons after OGD showed a shorter average length of the longest neurites and fewer primary neurites. Recent studies have showed that IL-10 directly protects cultured primary cortical neurons from OGD-induced injury in an anti-apoptotic way^12^,^14^. Our research shares and extends these findings. We noted that average length of the longest neurites and the number of primary neurites were significantly increased in the IL-10 group when compared with the OGD group. Moreover, we found that IL-10NA markedly decreased the average length of the longest neurites and the number of primary neurites. These results demonstrate that IL-10 promotes neurite outgrowth after OGD injury, suggesting that IL-10 plays a role in neuronal repair. Previous research has reported that activated JAK1 and STAT3 are up-regulated in neurons, astrocytes and microglia after focal cerebral infarction^21^,^22^. Furthermore, activated STAT3 promotes axon regeneration after nerve impairment in DRG and motor neurons^27^,^28^. However, it is still uncertain whether JAK1/STAT3 signaling pathway is directly involved in the effect of IL-10 on neurite outgrowth after OGD injury. Our data showed that JAK1 inhibitor GLPG0634 reduced the average length of the longest neurites and the number of primary neurites, indicating that JAK1/STAT3 pathway mediates the repair effect of IL-10 in cultured cortical neurons after OGD injury.

To explore the molecular mechanism by which IL-10 promotes neurite outgrowth after OGD injury, we analyzed the expression of Netrin-1. As one of canonical guidance cues, Netrin-1 not only participates in the neuronal development, but also plays a role in axonal outgrowth and branching^51^, neuronal survival^52^. A previous study indicated that expression of Netrin-1 was increased after stroke when detected by immunohistochemisty^53^. Furthermore, in our previous study, after the occurrence of cerebral ischemia, netrin-1 may play an important role in axon regeneration, forming new neural circuits by acting with DCC^54^. Indeed, another research demonstrated that Netrin-1 facilitates white matter repair and remodeling after focal cerebral ischemia^55^. Here, we presented for the first time that IL-10 up-regulated Netrin-1 expression in neurons after OGD injury, and IL-10NA reduced the expression of Netrin-1. What is more, IL-10-induced Netrin-1 expression was partly abolished by GLPG0634, suggesting that JAK1/STAT3 pathway mediates the effect of IL-10 on up-regulating the expression of the Netrin-1. To explore the role of Netrin-1, we examined the neurite outgrowth in IL-10 treatment after the Netrin-1 knockdown using shRNA. We found that both the average length of the longest neurites and average number of primary neurites were decreased in neurons transfected with Netrin-1 shRNA with or without IL-10 treatment, indicating that Netrin-1 mediates the effect of IL-10 on neurite outgrowth after OGD injury. Taken these results together, we demonstrate that IL-10 facilitates neurite outgrowth in cultured cortical neurons after OGD injury via JAK1/STAT3/Netrin-1 pathway.

As a biomarker of presynaptic plasticity and synaptogenesis^56^, synaptophysin facilitates axonal sprouting and synapse establishment, and ultimately encourages the recovery of neurological function after ischemia^8^. A recent study indicates that IL-10 directly protects cortical neurons by activating STAT3 signaling pathway^14^. Accordingly, activated STAT3 is reported to increase synaptophysin expression, which enhances synaptic plasticity and synaptogenesis in the hippocampus^29^. Another recent study shows that the administration of IL-10 promotes via the IL-10 receptor the synapse formation in cultured hippocampal neurons^15^. However, according to the literature available, it still awaits investigation whether IL-10 can promote synapse formation in neurons after OGD injury. In current study, we detected the expression of synaptophysin, the density of dendritic spines, excitatory and inhibitory synapses to ascertain whether IL-10 promotes synapse formation in cultured cortical neurons after OGD injury. We reported for the first time that IL-10 up-regulated the expression of synaptophysin, the density of dendritic spines, excitatory and inhibitory synapses in neurons after OGD injury and that these effects were partly reversed by JAK1 inhibitor GLPG0634. Taken together, we believe that IL-10 may promote synapse formation in OGD-injured primary cortical neurons through the JAK1/STAT3 pathway.

On the basis of the above analysis and discussion, the current study concludes that IL-10 not only attenuates neuronal apoptosis, but also facilitates neurite outgrowth and promotes synapse formation in cultured primary cortical neurons after OGD injury and that the neuroprotective effect of IL-10 on OGD-injured neurons is mediated by JAK1/STAT3 pathway and its down-stream targets Bax, Bcl-2, Netrin-1 and synaptophysin. Meanwhile, there are some limitations in our study: firstly, we only testified that IL-10 can facilitate neurite outgrowth and synapse formation *in vitro*, future studies should be done to verify its effect *in vivo*; secondly, the mechanism of the downstream molecule of Netrin-1 in promoting neurite outgrowth remains unclear. Nevertheless, the study extends our understanding of the neuroprotective effect of IL-10 and suggests its therapeutic potential against cerebral ischemia.

## Methods

### Animals

Pregnant Sprague Dawley rats were provided by the Animal Center of Fujian Medical University (Fuzhou, China). All animals were bred and housed under standard conditions. Efforts were made to reduce the number of animals used as well as their suffering and all experimental animals were euthanized with isoflurane which contained 3% induction, 1.5% maintenance in 30% O2 and 70% N_2_O. Experimental protocols followed the guidelines of the National Institute of Health (NIH Publications No. 80-23, revised in 1996) and were approved by Institutional Animal Care and Use Committee of Fujian Medical University.

### Primary Cortical Neuron Cultures

Primary cortical neurons were cultured as described previously with minor modifications^57^. Briefly, samples of cerebral cortex were prepared from brains of Sprague–Dawley rat embryos (aged 16–18 days). The cell suspensions were plated on coverslips (24 mm × 24 mm) with poly-L-lysine and cultured in a neurobasal medium (Gibco, USA) containing 2% B27 (Gibco, USA), 0.5 mM of glutamine and 50 U/ml of penicillin/streptomycin and the harvested neurons were cultured in chamber at 37 °C with 5% CO2. The neurobasal medium was firstly refreshed after 24 hours and then half of the medium was refreshed every three days. As a result, approximately 90% purity of neurons was obtained as determined by class III-β-Tubulin and Hoechst 33342 staining.

### Oxygen-Glucose Deprivation

Oxygen-glucose deprivation (OGD) model was established as described previously with minor modifications^57^. Briefly, seven days after plating, the primary cortical neurons were washed with glucose-free DMEM (Gibco, USA) and incubated with glucose-free DMEM. They were further incubated in an anaerobic chamber containing a mixture of 5% CO2 and 95% N2 at 37 °C for 90 minutes. Then, the cultures were switched back to their original culture condition for 24 hours or 48 hours. Neurons in the control group were treated without the OGD exposure.

### Drug Treatment

IL-10 from rat recombinant was purchased from PeproTech. It was administered to the cultured cortical neurons right before and after OGD, reaching a final concentration of 20 ng/ml. For neurons with IL-10, a specific inhibitor of JAK1, GLPG0634^30^ (SYNkinase, USA) was added to inhibit phosphorylation of JAK1 pharmacologically, reaching a final concentration of 20 nmol/L. IL-10 neutralizing antibody (IL-10NA, Abcam, UK) (5 μg/mL) was applied to cultured neurons right before and after OGD to antagonize the neuroprotective effect of IL-10. The experimental groups were designed as follows: control group, OGD group, OGD + IL-10 group and OGD + IL-10 + GLPG0634 group, OGD + IL-10NA group. Each group received equal volume of medium.

### Flow Cytometry Using Annexin V/PI Staining

To assess the apoptosis of neurons quantitatively, we performed flow cytometry as described previously with minor modifications^57^,^58^. In brief, cortical neurons were plated in culture flasks (25 mm^2^) following the experimental protocol described above. Twenty-four hours after OGD, the Annexin V/PI staining (Beyotime, China) was performed according to the manufacturer’s instructions. The neurons were resuspended and washed three times with 4 °C PBS. Next, the neurons were resuspended by 200 μL incubation buffer and then into the medium 5 μl of Annexin-V-FITC labeling reagent and 10μl of PI were added. The cells were then incubated at room temperature for 15 min in the dark. At least 1 × 10^4^ cells were recorded in each sample and were immediately analyzed using flow cytometry (Beckton Dickinson, USA). The experiment was repeated three times.

### Quantitative Reverse Transcription-PCR (qPCR)

Total RNA was isolated from cultured neurons using Trizol reagents (Life Technologies, USA) according to the manufacturer’s instructions. The quantity and purity of RNA were assessed by the ND-1000 micro-spectrophotometer (NanoDrop Technologies, USA). One microgram of purified RNA was reversely transcribed to cDNA using PrimeScript RT reagent Kit with gDNA Eraser (Takara, Japan). Quantitative real-time PCR was performed in the ABI Prism 7500 system (Applied Biosystems, USA) using SYBR Premix Ex Taq (Tli RNase H Plus) (Takara, Japan). The thermal cycling conditions were as follows: amplifications were performed starting at 95 °C for 30 seconds, followed by 40 cycles of 95 °C for 15 seconds and 60 °C for 34 seconds. Melting curve analysis began at 95 °C for 15 seconds, followed by at 60 °C for 1 minute and at 95 °C for 15 seconds. Specificity of the product amplification was confirmed by melting curve analysis. The primer sequences for each gene were as follows: rat Interleukin-10 Receptor 1 (IL-10R1), 5′-CACTGCTCCTCCGACCACTCT-3′ and 5′-CGGCATCATCTATGGGACAATC-3′; rat Bax, 5′-GCAGAGGATGATTGCTGATGTGG-3′ and 5′-AGGAAGTCCAGTGTCCAG-CCCAT-3′; rat Bcl-2, 5′-GATGACTTCTCTCGTCGCTACCGT-3′ and 5′-GGAGA- AATCAAACAGAGGTCGCAT-3′; rat Netrin-1, 5′-CTAAGCAGAACGAGCAGG-AGGCG-3′ and 5′-AGCACCGGCGAGTTGTCGAAGT-3′; rat synaptophysin, 5′-GCTGTGTTTGCCTTCCTCTACTCC-3′ and 5′-TTGATAATGTTCTCTGGGT- CCGTG-3′; rat GAPDH, 5′-AGGTTGCTTAAATGGCTCT-3′ and 5′-AACCCAGT- TTACCTCTTTG-3′. GAPDH was used as the reference gene and the expression of each targeted gene was analyzed by 2^−ΔΔCT^ method. Moreover, the control group was used as a calibrator sample and was set as 1× expression of each targeted gene. Three independent experiments were carried out.

### Western Blot Analysis

The expressions of IL-10R1, JAK1, p-JAK1, STAT3, p-STAT3, Bax, Bcl-2, Netrin-1 and synaptophysin from cultured neurons were analyzed by western blot after drug treatment. Briefly, cell extracts were obtained from primary cortical neurons using RIPA lysis buffer containing 1 mM PMSF (Beyotime, China). The supernatant was centrifuged at 14000 × g at 4 °C for 10 min. Concentration of the protein was determined by BCA kit (Beyotime, China). Equal amounts of total protein (20 μg) from every sample were separated by 10% or 12% SDS-PAGE and subsequently transferred to PVDF membranes (Millipore, USA). The membranes were blocked with blocking solution (Beyotime) for 1 h and then incubated with primary antibodies at 4 °C overnight: rabbit anti-IL-10R1, rabbit anti-JAK1 antibody, rabbit anti-pJAK1 antibody (Tyr1022/Tyr1023) (1:200, Santa Cruz, USA, respectively); rabbit anti-STAT3 antibody, rabbit anti-pSTAT3 antibody (Y705) (1:1000, Cell Signaling Technology, USA, respectively); rabbit anti-Bax antibody, rabbit anti-Bcl-2 antibody, rabbit anti-Netrin-1, rabbit anti-synaptophysin antibody and rabbit anti-GAPDH antibody (1:1000, Abcam, UK, respectively). After 3 washes with PBST, the membranes were incubated with goat anti-rabbit IgG-HRP secondary antibody (1:8000, Abcam, UK) at room temperature for 2 h and the signals of membranes were detected with ECL reagent kits (Beyotime, China). Band intensities were analyzed with the image J software (1.46r). The relative expression levels of proteins were normalized to the appropriate internal control. Three to five separate experiments were conducted.

### Transfection of Lentivirus

Five days after plating, cultured neurons were transfected with rat Netrin-1 shRNA lentivirus or with non-targeting scramble shRNA lentivirus (Genechem, Shanghai, China) at a MOI of 5 according to the manufacturer’s instruction. The target sequences of Netrin-1 shRNA were as follows: 5′-GCGACGAGAACGAAGATGA- 3′. Scrambled shRNA was used as a control and its sequences were as follows: 5′-TTCTCCGAACGTGTCACGT-3′. Forty-eight hours after transfection, cultured neurons were processed for various experiments.

As described previously with minor modifications^15^, 7 days after plating, cultured neurons were transfected with lentivirus containing the gene for green fluorescent protein (GFP). Twenty-four hours after transfection, cultured neurons were processed for OGD and drug treatment. After 7 days, at DIV 15, the neurons were taken to assess the density of dendritic spines and synaptic puncta.

### Immunofluorescence Staining

Forty-eight hours after the drug treatments, imunofluorescence staining was performed to evaluate the neurite outgrowth and locate the expression of Netrin-1 as described previously^54^,^57^. In addition, at DIV 15, the density of dendritic spines and synaptic protein clusters of the cultures were measured by immunofluorescence staining. Neurons were washed with PBS for three times and then fixed in 4% paraformaldehyde (pH 7.4) for 15 minutes. Cells were incubated overnight at 4 °C with the following primary antibodies: rabbit anti-IL-10R1 antibody (1:300, Santa Cruz, USA), rabbit anti-Netrin-1 antibody (1:200, Abcam, UK), rabbit anti-GFP antibody (1:1000, Abcam, UK), mouse anti-vGLUT antibody (1:200, Abcam, UK), anti-vGAT antibody (1:50, Santa Cruz, USA) and mouse anti-class III-β-Tubulin antibody (1:400, Beyotime, China). After 3 washes with PBS, they were further incubated with corresponding secondary antibodies (Cy3 donkey anti-mouse IgG, at 1:400 and Dylight488 donkey anti-rabbit IgG, at 1:800, Jackson Immunoresearch, USA, respectively) at room temperature for 2 hours. Nuclei were stained with Hoechst33342 (5 μg/ml, Sigma, USA). Glass slides were viewed in a ZEISS LSM 780 confocal microscope (Carl Zeiss, Germany), and the length of longest neurites and number of primary neurites which directly originated from the soma were quantified by LSM Image Browser (V4.2.0.121). For each group and experiment, 20 neurons from 8~10 randomly selected fields were observed to assess the average length of the longest neurites and average number of primary neurites. For each group and experiment, the density of dendritic spines (0.4–2.5 μm) and synaptic puncta were measured from 20 dendrites of eight to ten neurons and the total dendritic length of ~50 μm was measured from the first dendritic branching points by using ImageJ software as described previously^15^,^59^,^60^. All trials were repeated three times.

### Statistical Analysis

Data were expressed as Mean ± SEM and analyzed by SPSS19.0 statistical software (IBM, USA). Three to five independent experiments were carried out for all measurements. Statistical significance among groups was determined by one way analysis of variance (ANOVA) followed by Student-Newman-Keuls multiple comparisons test when equal variances were assumed. When equal variances were not assumed, Dunnett’s T3 was applied. The significance of mean differences between two groups was calculated by unpaired two-tailed Student’s t-tests. P values less than 0.05 (two-sided) were considered as statistically significant.

## Additional Information

**How to cite this article**: Chen, H. *et al*. IL-10 Promotes Neurite Outgrowth and Synapse Formation in Cultured Cortical Neurons after the Oxygen-Glucose Deprivation via JAK1/STAT3 Pathway. *Sci. Rep.* **6**, 30459; doi: 10.1038/srep30459 (2016).
